# Chromatin Fiber Dynamics under Tension and Torsion

**DOI:** 10.3390/ijms11041557

**Published:** 2010-04-12

**Authors:** Christophe Lavelle, Jean-Marc Victor, Jordanka Zlatanova

**Affiliations:** 1 Interdisciplinary Research Institute, CNRS USR 3078, Villeneuve d'Ascq F-59655, France; 2 Laboratoire de Physique Théorique de la Matière Condensée, CNRS UMR 7600, Paris F-75005, France; 3 Department of Molecular Biology, University of Wyoming, Laramie, WY 82071, USA

**Keywords:** DNA, nucleosome, chromatin, single molecule, magnetic tweezers, optical tweezers

## Abstract

Genetic and epigenetic information in eukaryotic cells is carried on chromosomes, basically consisting of large compact supercoiled chromatin fibers. Micromanipulations have recently led to great advances in the knowledge of the complex mechanisms underlying the regulation of DNA transaction events by nucleosome and chromatin structural changes. Indeed, magnetic and optical tweezers have allowed opportunities to handle single nucleosomal particles or nucleosomal arrays and measure their response to forces and torques, mimicking the molecular constraints imposed *in vivo* by various molecular motors acting on the DNA. These challenging technical approaches provide us with deeper understanding of the way chromatin dynamically packages our genome and participates in the regulation of cellular metabolism.

## 1. Introduction

**Figure 1.**
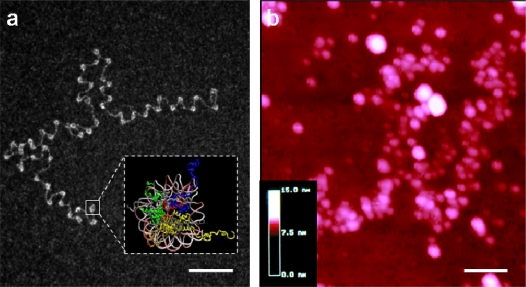
Molecular microscopy views of chromatin fibers. (**a**) Transmission Electron Microscopy (TEM) image of a nucleosomal array extracted from Chinese hamster ovary cells, spread in water and observed in annular darkfield mode after uranyl acetate staining (bar 100 nm); adapted from [7]. Insert: nucleosome crystal structure (from 1kx5 PDB coordinates). (**b**) Atomic Force Microscopy (AFM) image of unfixed chromatin fibers extracted from chicken erythrocytes and spread on glass in low ionic strength buffer (imaged area 600 × 600 nm); adapted from [8].

A primordial question in biology today is: how does DNA regulate its own metabolism in general, and transcription in particular? In other words, where is the regulatory information that enables specific genes to be transcribed while others are silenced? Chromatin inevitably holds a significant part of the answer. DNA is indeed not naked *in vivo* but is associated, mainly with histones, to form a nucleoprotein complex whose structure and dynamics participate in the regulation of various DNA transaction events, including replication, transcription, repair and recombination (Figure 1). Single-molecule approaches have recently complemented conventional biochemical and biophysical techniques and appeared as powerful tools to decipher the complex mechanisms ruling chromatin function and dynamics [1–3]. In these approaches, individual nucleosomes or chromatin fibers are investigated one-at-a-time through manipulation with different micro-systems. Here, we will mainly focus on results obtained via magnetic tweezers (MT) and optical tweezers (OT) [4]. Thanks to these techniques, we now have access to dynamic events that tend to be blurred when using traditional biochemical bulk experiments [5,6]. Furthermore, we can study one molecule at-a-time, thus gaining insights into the behavior of non-homogeneous specimens, whereas usual biochemical and biophysical techniques measure only the average behavior of the members of a population.

Since this field is quite recent, we will try to draw an almost exhaustive landscape of the many insights into chromatin structure and dynamics that were obtained by these techniques. Note, however, that for the scope of this focused review, we will only describe papers dealing with “conventional” chromatin (*i.e.*, DNA compacted by histones), although fascinating results have also been obtained with other DNA-condensation mediators such as protamine in sperm chromatin [9], Abf2p in mitochondrial chromatin [10], or HU and H-NS in bacterial chromatin [11,12]. With the growing popularity of single-molecule approaches, more and more labs should soon be equipped with sophisticated instrumentation enabling investigation of structure, dynamics, forces and motions at the same time [13,14]. There is no doubt that the chromatin field has, and will further, benefit from this trend, hopefully uncovering some of the remaining mysteries of this “DNA manager”.

## 2. Nanotweezers: A New Tool to Handle Macromolecular Complexes

Since first introduced to biological research about 15 years ago, single-molecule manipulation techniques have progressively broadened their application range and appear now as sensitive and versatile tools to study molecular mechanisms [5].

**Figure 2.**
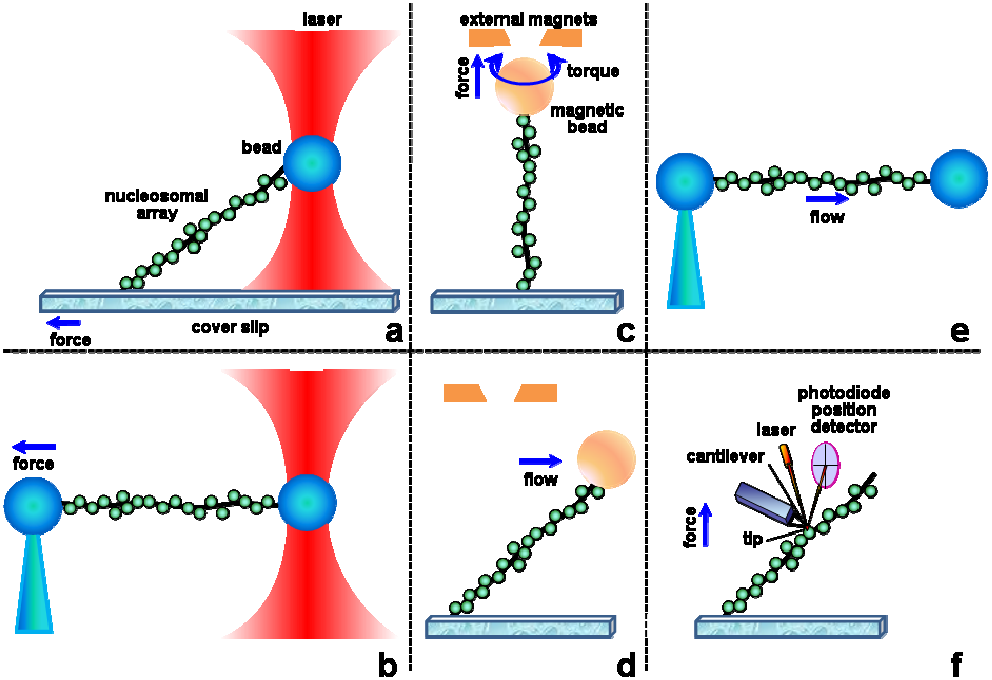
Various methods have been developed to manipulate single nucleosomal arrays. Depending on the setup, these experiments enable the study of nucleosome assembly/disassembly under constraints, to apply force (tension) and/or torque (torsion) on chromatin fibers and measure their mechanical response. These techniques use either OT (**a**,**b**), MT (**c**,**d**), flow (**d**,**e**) or the cantilever of an AFM (**f**) to apply constraints to a chromatin fiber attached at the other end to the surface of a cover slip (a,c,d,f) or to the extremity of a micropipette (b,e). Chromatin can thus be pulled (a,b,c,d,e,f) and rotated (c). a, b, and f are position clamps, while c, d, and e can be used as both force or position clamps.

Biologically relevant forces vary from picoNewtons (pNs) (thermal fluctuation, entropic forces) to tens of pNs (produced by some powerful molecular motors, such as RNA polymerases). Molecular bonds span a few pNs for weak bonds (van der Waals, hydrogen and ionic bonds) to hundreds of pNs for covalent bonds. Note that antigen/antibody interaction forces are estimated at 100–250 pN [15], even though they rely only on weak bonds (working in parallel). Various tools have been developed that can exert and/or measure such forces at the single-molecule level, mainly mechanical force transducers (atomic force microscope cantilevers, microneedles, optical fibers) and external field (photonic in optical tweezers, magnetic in magnetic tweezers, hydrodynamic in flow field systems) manipulators [1,16–18] (Figure 2). In typical experiments, one investigates DNA or nucleosomal templates one-at-a-time, either by physically exerting forces and measuring subsequent deformation of the substrate or by using DNA as a mechanosensor to measure DNA-protein interactions. These devices are of two different kinds: position clamps, where one imposes the position of the object and measures the force needed to do so (AFM, microneedles and optical tweezers) and force clamps, where a constant force is applied and the extension of the molecule is measured (magnetic tweezers, hydrodynamic flow). Note that AFM and OT can be operated in both modes, the introduction of a feedback enabling them to work as force clamps. Results obtained with optical and magnetic tweezers, the two most used systems, will be discussed here in more detail.

### 2.1. Optical Tweezers (OT)

Optical tweezers use a focused laser beam to physically hold and move microscopic dielectric beads. Ashkin and colleagues reported the first use of what is now commonly referred to as an optical trap: a tightly focused beam of light capable of holding microscopic particles stable in three dimensions [19]; see [20] for a recent overview of the physical principles behind optical tweezers. In the late 1980s, Ashkin and colleagues first applied the technology to the biological sciences, using it to trap individual tobacco mosaic virus particles, *Escherichia coli* bacteria and various eukaryotic cells [21,22]. Throughout the 1990s and afterwards, researchers like Carlos Bustamante and Steven Block pioneered the use of optical trap force spectroscopy to characterize molecular-scale biological motors [23]. The first application to chromatin fibers appeared in 2000 [24] and other insightful experiments rapidly followed (reviewed in [25,26]). “Chromatin optical tweezers” in PubMed now retrieves 20 references, only half of which are primary experimental papers actually dealing with nucleosomes or chromatin fibers.

### 2.2. Magnetic Tweezers (MT)

Magnetic tweezers use external magnets to physically pull/rotate microscopic paramagnetic beads to which one attaches DNA molecules or chromatin fibers. Typical applications are single-molecule micromanipulation and studies of force/torque-regulated processes. Forces are typically on the order of a few piconewtons. Due to their simple architecture, magnetic tweezers are one of the most popular and widespread biophysical techniques [27]. They were first used to handle a single DNA molecule and measure its elasticity [28] and response to torsion [29], before being applied to study single nucleosomal arrays [30,31]. “Chromatin magnetic tweezers” in PubMed now retrieves 15 references, of which a third are primary experimental papers actually dealing with chromatin fibers.

## 3. Chromatin Fiber Manipulation

Tweezers provide a powerful technique to test the response of chromatin fibers to physiological levels of tension and/or torsion; the results from these studies greatly improved our knowledge of nucleosome structure and dynamics within chromatin fibers [32]. Table 1 summarizes the 25 or so papers published so far that address nucleosomal fiber dynamics using either flow forces, OT or MT experiments. These experimental approaches (and the use of the AFM) to manipulate fibers are depicted in Figure 2.

### 3.1. Chromatin Assembly/Disassembly

The first single-molecule study of chromatin assembly was carried out by Viovy's group [33]. They used real-time video-microscopy to follow the shortening (as a consequence of DNA wrapping around the histone core that occurs during nucleosome formation) of a single λ-DNA molecule attached at one end to a glass surface, the other end being stretched by the flow of cell-free extracts in the chamber. Other approaches to address chromatin assembly have been setup by Bennink *et al*. [36] (Figure 2e) and Leuba *et al*. [30] (Figure 2d) by attaching a bead to the free extremity of the DNA/chromatin fragment for subsequent manipulation of the assembled fibers. These studies showed that the kinetics of assembly is dependent on the applied force; nucleosome formation is inhibited at forces >10 pN.

Recently, nucleosome assembly has been studied on topologically-constrained DNA to more closely mimic the *in vivo* situation, where chromatin fibers are topologically constrained in loops [57]. Assembly was achieved using chicken erythrocyte core histones and histone chaperone Nap1. Only partial assembly was observed on the topologically-constrained DNA tethers, with much more complete assembly on unconstrained (nicked) DNA. To verify the hypothesis that the lack of full nucleosome assembly was due to compensatory accumulation of positive supercoiling in the rest of the template, experiments were performed in which the positive stress was mechanically relieved by rotating the external magnetic field; indeed, such rotation led to resumption of assembly. The positive supercoiling density that led to stalling of assembly was estimated to be in the range of 0.025–0.051.

Chromatin disassembly under applied force has been mostly studied using optical tweezers [24,35,37,40,41] (Figure 2a,b) and only more recently with magnetic tweezers [31] (Figure 2c). Note that the first pulling studies on single chromatin fibers were done by AFM [58,59], but it turned out to be quite tricky to interpret due to interactions of the nucleosomes with the glass surface [4]. Indeed, saw-tooth patterns were observed in force-distance curves, similar to those expected from unravelling of individual nucleosomes (and later seen in OT experiments, see below). Careful analysis of the data, however, revealed that the drops in force observed were simply due to detachment of individual nucleosomes from the surface of the AFM cells [4]. Note that getting rid of such surface interaction artefacts is also one of the major issues in OT and MT experiments, particularly when playing with histones, which are highly positively charged, and thus very sticky to surfaces. One should be careful to check that discrete signals such as saw-tooth patterns or steps are not due to nucleosome desorption from the surface of the beads; this can usually be done by repeating the experiments at various forces and in various salt conditions, and also by adding naked DNA spacer at each end of the reconstituted nucleosome array (to prevent histone sticking to surfaces; see below). Finally, correspondence between the number of steps and the number of nucleosomes on one hand, and the step size and the nucleosomal DNA length on the other hand, is a good indication of the relevance of the experiment [31,35,37].

**Figure 3.**
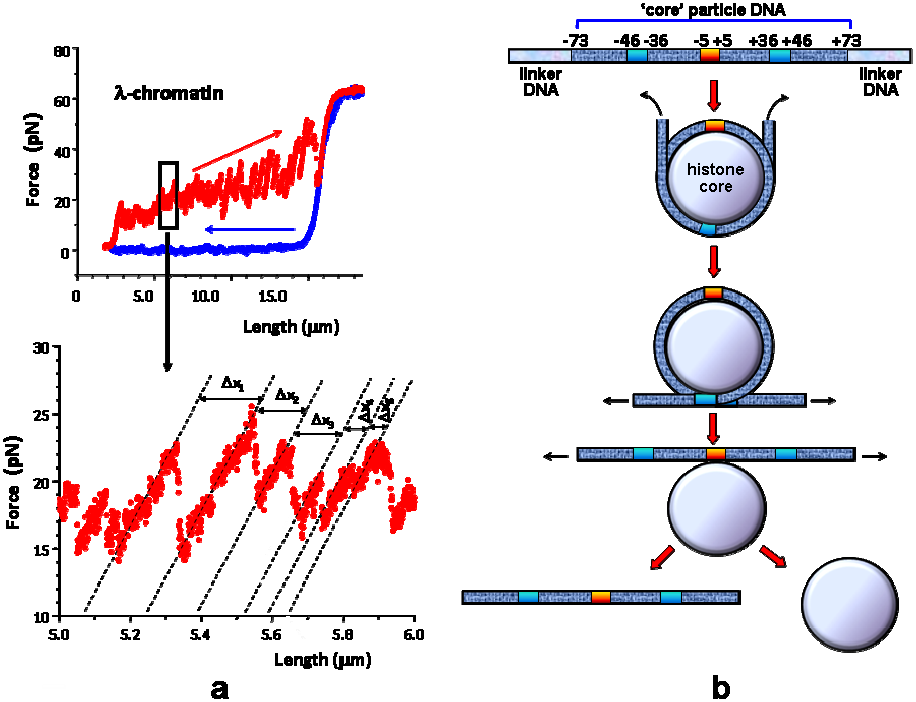
Force/extension curves and their interpretation (see experimental setup Figure 2a). **(a)** Force *versus* length response of a single chromatin fiber handled by an OT. The fiber was directly assembled in the flow cell of the instrument from a single λ DNA molecule and *Xenopus* cell-free extracts: these contain all core histones but lack linker histones. A portion of the representative force curve (upper panel) is enlarged in the lower panel. The discontinuities in the curve correspond to unraveling of individual nucleosomes within the fiber. The blue curve represents the relaxation) response, which exhibited a naked DNA-like behavior [35]. **(b)** A schematic of the step-wise mechanical disruption of the nucleosomal particle (unpeeling of nucleosomal DNA from the histone core) as suggested by Brower-Toland *et al*. [37] (see also Table 1).

Force measurements in OT setups have revealed the existence of internucleosomal attraction that maintains the compacted chromatin structure under physiological condition [24]. These studies showed that, in the absence of any chromatin remodeling factors, the fibers undergo reversible stretching at forces <∼20 pN and irreversible unfolding at forces >∼20 pN [35,37,40]. A step-wise release of the DNA/histone interactions was observed by Wang and colleagues [37] (Figure 3) and recently confirmed on single mononucleosomes [44]. Various chromatin remodelers use the energy of ATP hydrolysis to facilitate the process *in vivo*, lowering the unfolding energy barriers [31,41]. Finally, it was recently possible to measure, in DNA unzipping experiments, the relative strengths of the histone/DNA interactions along the length of nucleosomal DNA [47].

### 3.2. Chromatin Topology

**Figure 4.**
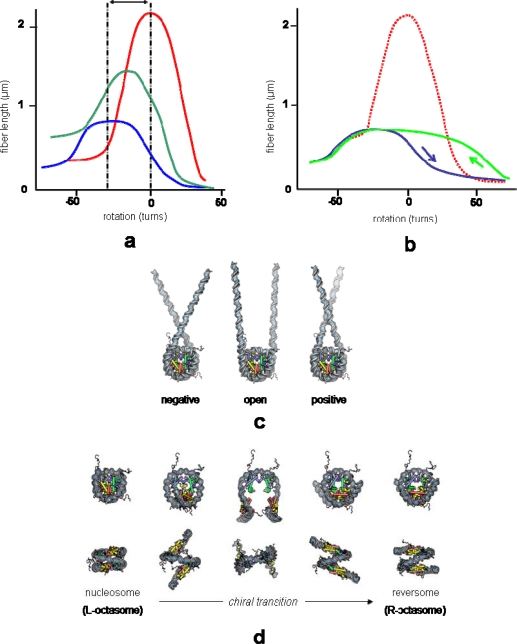
Rotation/extension curves and their interpretation (see experimental setup in Figure 2c). (**a**) Length *versus* rotation response at 0.35 pN of naked DNA (red), partially reconstituted (green) and saturated (blue) nucleosomal array. (**b**) Length *versus* rotation response of a saturated reconstituted nucleosomal array. Hysteresis is observed between the onward (blue) and backward (green) curves when a high positive torsion is applied [up to 70 positive turns, while torsion applied was less than 50 turns in (a)]; the zero-turn rotation reference corresponds to the relaxed state of naked DNA (red dotted curve). (**c**) The shortening, shifting and flattening of the curves in (a) is interpreted as the consequence of nucleosome reconstitution (each nucleosome wraps ∼50 nm of DNA in one negative superhelical turn) and conformational flexibility (three-state model) [31]. (**d**) The hysteresis observed at high torsion in (b) is interpreted as the consequence of a transient chiral transition of nucleosomes to an altered right-handed form [53].

Three years after Leuba/Zlatanova's MT studies of chromatin assembly, Bancaud *et al*. succeeded in using MT to apply torsion to single chromatin fibers, showing that chromatin can accommodate surprisingly large amount of torsional stress, either negative or positive, without much change in its extension [31,53]. In these two studies, nucleosome arrays were reconstituted on 2 × 18 tandem repeats of the 208 bp 5S nucleosome positioning sequence. They were subsequently ligated at each end to a naked DNA spacer (introduced into the construct to prevent histone sticking to surfaces) followed by a DNA sticker labeled appropriately for attachment of one end of the construct to the coated bottom of the flow cell and of the other end to a paramagnetic bead (Figures 2c). The rotation of the magnets, hence of the paramagnetic bead, exerts torsion on the attached fiber. The fiber torsional behavior is described, at a given force, by its length-versus-rotation plot (Figure 4a).

The response of the corresponding naked DNA, obtained following salt dissociation of the histones, displays the signature of an intact (non-nicked) single duplex DNA. The central (plateau-like) part of the DNA response curve corresponds to the elastic regime, and the quasi-linear compactions on either side to the plectonemic regimes (formation of positive or negative plectonemes upon introduction of positive or negative rotations). The lower compaction on the negative side is due to a force-dependent DNA melting at high negative torsions, which prevents further increase of the torque on the molecule and prevents further plectoneme formation. Compared to DNA, chromatin is shorter and its center of rotation is shifted to negative values (see double arrow in Figure 4a). The shift is the expected consequence of the absorption of approximately one negative superhelical turn per nucleosome (actually Δ*Lk_p_* ≈ −0.8 ± 0.1; see below), the observed shortening (∼50 nm, *i.e.*, 150 bp per nucleosome) resulting from DNA wrapping around the histone core. Therefore, compared to DNA of the same length, the fiber in the elastic regime appears to be extremely torsionally flexible, *i.e.*, it can absorb large amounts of torsion without much shortening. This large torsional resilience was interpreted as a reflection of the nucleosome dynamic equilibrium between the three conformational states previously identified in minicircles studies [60–62] (Figure 4c). More surprisingly, these chromatin fibers, after extensive positive supercoiling, display no nucleosome loss but rather a hysteretic behavior in their mechanical response to torsion [53] (Figure 4b). This hysteresis was interpreted as a consequence of the trapping of positive turns in individual nucleosomes through their transition to an altered form, originally called reversome (for *rever*se nucleo*some*) and later termed R-octasome (for right-handed nucleosome containing the full complement– 8–of histones) [63]. This structure is reminiscent of the previously documented R-tetrasome (a right-handed sub-nucleosomal particle containing only the H3/H4 histone tetramer), which occurs as a result of a chiral transition of the tetrasome [64] (Figure 4d).

### 3.3. Chromatin Mechanical Parameters

To better characterize how the chromatin fiber may respond to mechanical constraints, some elastic parameters have to be determined, including bending flexibility, torsional flexibility and stretching modulus. Micromanipulation approaches coupled with modeling have been crucial in estimating these parameters both for DNA and chromatin. However, one should keep in mind that, beside divergences arising from experimental and data analysis differences, DNA sequence effects [65] and geometrical variability [66,67] can greatly influence DNA and chromatin elastic parameters, respectively. Hence, the given values are mostly to be taken as orders of magnitude (Table 2). It is also worth noticing that, whereas forces are easily and precisely evaluated by OT as well as MT, torques remained more difficult to access [68,69]. The two devices can indeed be used to twist the molecule: in optical tweezers, light polarization is used to apply a torque, while in magnetic tweezers the magnetic field imposes the angular position of the molecule. In both cases, the torque in the range of pN.nm is far more difficult to measure than the stretching force. Remarkably, Sun and colleagues recently succeeded in measuring the torque exerted on a chromatin fiber by using a new MT setup that manipulates a ferromagnetic rod coupled to a magnetic bead in the field of a cylindrical magnet [70].

The persistence length of a polymer, A, a measure of bending flexibility (the smaller A, the more flexible), has been determined for DNA in various experiments, leading to a consensus value of ∼50 nm (150 bp) at physiological ionic strength [71,72]. The values for chromatin are more divergent, from ∼ 30–50 nm estimated from single-molecule stretching [24,31,35], recombination frequencies [73] or cross-linking probabilities [74] to more than 200 nm from recent *in situ* hybridization experiments [75]. Remarkably, such different values are all consistent with theoretical models and could be explained by polymorphic connection geometry between nucleosomes in the fiber [66,67].

The torsional persistence length C, a measure of twisting flexibility, has been estimated for naked DNA by various bulk experiments to be ∼70–100 nm (200–300 bp) [76,77]; this value is consistent with single-molecule data [29] analyzed through an elastic model [78]. The torsional persistence length of the chromatin fiber, measured only recently, has a surprisingly low value of 5 nm [31]; this is almost 20-times lower than that of naked DNA, and also lower than the value predicted from analytical models of chromatin fibers [66]. However, this unexpected experimental result is easily explained by the conformational nucleosome dynamics (see discussion above), and is fully consistent with new chromatin models that include this nucleosome property [31].

The stretching modulus σ, a measure of stretching elasticity (*i.e.*, the propensity of a polymer to reversibly extend under a given tension - the smaller σ, the more extensible), is ∼1100 pN for naked DNA, as estimated from single-molecule experiments [79–81]. For the chromatin fiber, a much lower value of ∼ 5–8 pN has been derived from the same kind of experiments [24,31]; such value is close to the predicted one [66] and probably reflects the flexibility of the linkers as well as the weak internucleosomal interactions disrupted during the mechanical extension of the fiber at low ionic strength. Confirming this view, the stretching modulus is indeed greatly enhanced in high salt conditions where the fiber adopts a more compacted state [35,82].

### 3.4. Fiber Structure

Whereas MT and OT provide a unique tool for probing the mechanical properties of DNA-protein complexes, in particular those of nucleosomes, they remain of little help in characterizing chromatin fiber structure. As a matter of fact, force-extension diagrams require an underlying model for their interpretation. Of note, the Hookian behavior of regular arrays of nucleosomes, recently evidenced by van Noort and colleagues, has been claimed by this group to match a solenoid structure for a 197-bp repeat length array structure but a zig-zag structure for 167-bp fiber [55]. In their model, they assumed *a priori* a solenoid structure for the 197-bp repeat length array from which they deduced a nucleosome-nucleosome stacking energy as large as ∼14 kT, despite the fact that nucleosomes are loosely stacked in their solenoid structure. This stacking energy is obtained by fitting the force-extension plots with a mechanical model involving eight parameters, five of which are free. In this model, the Hookian behavior is related to the elasticity of the disordered histone tails which are assumed to bridge the stacked nucleosomes, until they eventually break at forces >3 pN in absence of linker histone.

At odds with this modeling, an alternative interpretation has been proposed (Victor *et al*, submitted) that assumes a zig-zag structure for both 167-bp and 197-bp fibers [83,84]. In this model, the Hookian behavior is related to a gradual unwrapping of the nucleosomal DNA, by consecutively breaking the histone/DNA contacts at the Super Helix Locations: SHL ± 6.5, then SHL ± 5.5, and eventually SHL ± 4.5. Note that the Hookian behavior as well as the corresponding spring stiffness of the fibers, either 167-bp or 197-bp, are well fitted with the so-called “tunable spring” model [66], in which the fiber stiffness is completely determined by the fiber geometry and the linker DNA persistence length. There is only one free parameter, namely the free energy of the SHL ± 5.5.

### 3.5. Chromatin Remodeling

Remodeling has been also addressed using MT [46] and OT [43,45] (see [85] for a recent review). To date, only one family of remodelers has been studied by single-molecule manipulations, specifically the *S. cerevisiae* RSC and SWI/SNF complexes of the Swi2/Snf2 family. These remodelers are ATP-dependent DNA translocases. They contain two DNA-binding domains: one stationary tracking domain which remains in a fixed position relative to the histone octamer, and the other alternating between one of two conformations. The two domains bind the DNA at two different sites on the duplex, stabilizing a transient DNA loop, resulting in a temporary shortening of the molecule. Note that this shortening is what is monitored by MT. The inchworm-like action of both domains imparts DNA translocation.

The first single-molecule observation of DNA translocation by a remodeler has been performed in the group of Vincent Croquette and David Bensimon in collaboration with Tom Owen-Hughes [46]. Using MT with naked DNA, they observed at forces <1 pN that a single RSC complex causes transient shortening of the DNA, which results from the formation of a negatively supercoiled loop. At the same time, Bustamante's group used OT to monitor the action of both RSC and SWI/SNF complexes on single nucleosomes in real time [45]. This group worked at forces >3 pN in order to avoid loop formation within bare DNA; in this way they could study the translocase activity specifically related to nucleosome remodeling in isolation. Their setup gives access to the physical parameters associated with translocation except for the twist, namely speed, force and processivity. Importantly, DNA translocation has been observed to occur at constant speed under varying forces, suggesting that remodelers function as motors rather than as ratchets [76].

**Figure 5.**
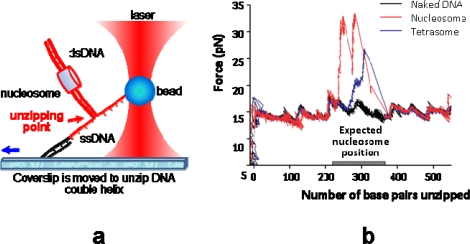
Nucleosome remodeling assessed by OT. (**a**) The nucleosome construct is attached to the bead by a biotin-streptavidin bond and to the coverslip by a digoxigenin--anti-digoxigenin linkage. The bead is kept fixed by tuning the laser power (optical trap) while coverslip is moving away from the bead, imparting the unzipping of the (in red) part of the dsDNA from the nick. (**b**) Plotting applied force (calibrated by laser power) *versus* number of base pairs unzipped provides a mapping of nucleosome position at single base-pair resolution. The three curves are the results obtained with three different constructs: naked DNA (in black), tetrasome (in blue) and nucleosome (in red). After remodeling by SWI/SNF the nucleosome is moved away from its initial position. Repeating the experiment on a sample of ∼150 nucleosomes results in a histogram of displacements, that is symmetrical around zero with a standard deviation of 28 bp.

Wang's group recently used a sophisticated “unzipping” technique to analyse single nucleosome products after remodeling by SWI/SNF [43] (see Figure 5 for details). The general mechanism that emerges from these single-molecule studies is a “DNA inchworm” model involving both twist and loop propagation (∼1 bp of twist for 10 bp of translocation) [85]. This model explains why the displacements after remodeling that are measured by Wang's group are much smaller than the loop sizes measured by the other two groups. Indeed, the inchworm-like action of both DNA-binding domains generates large loops that are likely to be resorbed substantially before imparting DNA translocation. As a result, the displacements are expected to be much smaller than the loop sizes. Single-molecule studies with the three other families–Iswi, Nurd/Mi-2/Chd and Ino/Swr1–are anticipated, with a special attention to the ISWI complexes.

## 4. Conclusion and Perspectives

More than providing a “natural barrier” to DNA accessibility and compacting DNA, chromatin has functional roles that relevant experiments and models should help us to understand. Chromatin faces electrostatic, elastic and topological constraints that have to be integrated in multiscale models including both structural and dynamical parameters. The three-dimensional chromatin structure depends on distinct but highly coupled parameters: DNA sequence, nucleosome spacing (and the regularity of this spacing), histone modifications (through incorporation of histone variants and/or post-translational modifications), nucleosome conformation (through DNA fluctuations at the entry/exit sites and potential deformation of the core particle itself), interactions within the fiber (through histone tail interactions and DNA elasticity and topology) and non-histone proteins possibly present (*i.e.*, HMG proteins, HP1 in heterochromatin, TRF1/2 in telomeres). Only recently, these issues are being addressed by single-molecule techniques, as seen from the comprehensive list of references in Table 1; 22 of the 28 total are less than five years old.

So, what did we learn from these studies that we did not know before? First, from a quantitative point of view, we got direct measurements of internucleosomal interactions, DNA/histone binding and unbinding steps, forces and torques produced by molecular motors acting on DNA (and how differently these motors behave on a chromatin template) and the response of chromatin fibers to these mechanical constraints. From a qualitative point of view, two main features should be retained from these studies: first, torsional and bending deformations are transmitted at about the same distances in DNA but not in chromatin, where torsional flexibility may be as much as 40 times higher than bending flexibility. Secondly, the stretching modulus being much greater (up to 200 times) for DNA than for chromatin, tension applied by a molecular motor on the DNA backbone will cause chromatin distortion well before DNA distortion. These features are undoubtedly valuable to understanding the function of chromatin *in vivo*. One should also acknowledge some frustrating limitations of these studies: so far, OT and MT experiments greatly improved our knowledge of nucleosome structure and dynamics within chromatin fibers, but failed to reveal chromatin structure/folding itself (e.g., the enigmatic and highly debated 3D structure of the so-called 30nm fiber).

Where should we go now? Chromatin is a polymorphic substrate, comprising nucleosomes and their more or less specific interactions influenced by DNA topology. Time has come to transpose the mechanistic approach to DNA considered as a “molecular spring”–which has proved valuable for the study of many biological processes, including DNA/protein recognition, nucleosome positioning and transcription initiation–to the next and more physiological structural level, *i.e.*, the chromatin fiber. From a purely mechanistic point of view, the chromatin fiber may be regarded as a complex and polymorphic functional “supramolecular metaspring” (basically, a spring–the chromatin fiber–made of loops and spirals of another spring–the DNA molecule) [66]. In the quest of mechanistic understanding of transcriptional regulation, a detailed description of the nuclear architecture, with the function of its subcompartments, and of the genome organization is obviously required, along with the extent to which nuclear structural proteins (lamin, actin) influence higher-order chromatin structure and tissue-specific gene expression [32]. For this, single-molecule fluorescence spectroscopy [86] along with single-molecule manipulation *in vivo* [87,88] is expected to provide exciting outcomes. At the same times, enzymatic processes (topological relaxation, transcription, DNA processing by molecular motors), that have been thoroughly studied on naked single DNA molecules, need to be investigated on their physiologically relevant substrate - single chromatin templates. The accumulation of new quantitative data (both *in vitro* and *in vivo*) and molecular models provided by these complementary approaches should help to drive us towards a more consistent and biologically relevant mechanistic view of genetic expression within the next years. The technological advances we have been witnessing during the past decade open new and exciting venues for research.

## Figures and Tables

**Table 1.** A comprehensive outline of published chromatin single-molecule manipulations. This table spans roughly 10 years (and about 30 papers, in chronological order within each category) of nano-handling of individual nucleosomes or chromatin fibers.

**Ref.**	**Approach**	**Observation/conclusion**
***Flow force experiments***		
[33]	Fluorescence video-microscopy was used to follow chromatin assembly on individual DNA molecules immersed in a cellular extract (Figure 2d, without the bead at the end of DNA).	DNA molecules were compacted within a few seconds into fibers resembling native chromatin fibers (as assessed by AFM imaging).
[34]	Kinetics of assembly on single DNA molecules were studied by fluorescence video-microscopy in the presence of either *Xenopus* egg extracts or purified histones with chaperone Nap1 (Figure 2d, without the bead at the end of DNA).	The assembly rates differed by a factor of up to 1000 for the same amount of histones depending on the system used. Faster kinetics and higher packing ratios were reached with extracts, presumably indicating a role of additional components present in this system.

***Optical Tweezers (OT) experiments***		
[24]	OT were used to stretch single chicken erythrocyte chromatin fibers (Figure 2b).	A reversible condensation-decondensation transition appeared at 5–6 pN. This corresponds to an internucleosomal attraction energy of ∼3 kT, suggesting that the fiber can interconvert between open and closed states at physiological ionic strength just because of thermal fluctuation. At forces >20 pN, the fibers were modified irreversibly, probably because of removal of nucleosomes from the DNA template.
[35,36]	λ phage DNA molecules were suspended between two polystyrene beads, one held by a micropipette and the other one by an optical trap (Figure 2b). Chromatin was assembled using *Xenopus* egg extracts, which was removed before stretching.	DNA apparent shortening revealed chromatin assembly on the DNA template; assembly was impeded by forces >10 pN. Stretching of the assembled chromatin fiber at forces >20 pN revealed sudden drops in force reflecting discrete opening events of ∼65 nm length, attributed to unwrapping of nucleosomes (Figure 3).
[37]	OT were used to stretch nucleosomal arrays reconstituted on DNA fragments containing 17 direct tandem repeats of a 5S sequence (Figure 2a).	Forced disassembly of each nucleosome occurred in three stages, corresponding to the successive unwrapping of the two turns of DNA followed by complete loss of the histone octamer (Figure 3).
[38]	OT were used to examine the contributions of histone tails to nucleosomal stability (Figure 2a).	Enzymatic removal of histone tails as well as their acetylation weakened the histone-DNA interactions leading to partial DNA unwrapping.
[39]	OT were used to stretch native and reconstituted nucleosomal arrays at various concentrations (Figure 2b).	Stretching a single chromatin fiber in very dilute solutions showed ∼25 nm discrete disruption length (interpreted as H2A-H2B dimers release), whereas a second ∼50 nm length was observed at high chromatin concentration (interpreted as full nucleosome disruption). These results demonstrate that nucleosome stability highly depends on experimental conditions (sample concentration).
[40]	OT were used to stretch reconstituted chromatin fibers (Figure 2b).	Fiber length increase per unbinding event showed discrete values of ∼30 nm and ∼60 nm. Loading rate analysis of the disruption forces revealed three individual energy barriers (∼20, 25 and 28 kT), with no apparent correlation with DNA length release.
[41]	OT were used to monitor nucleosome disassembly from a regular nucleosomal array preassembled in the presence of Nap1 and chromatin remodeling factor ACF (Figure 2b).	Abrupt events releasing ∼55–95 bp of DNA were observed at a wide range of unravelling force (∼5–65 pN), suggesting a strong dependence on the DNA sequence within individual nucleosomes. This variability in nucleosomal strength and the occurrences of sudden DNA re-wrapping events is thought to have an important regulatory influence on the binding of transcription factors and the movement of polymerase complexes on chromatin.
[42]	Chromatin fibers purified from HeLa cells were tethered between a microscope coverslip and a glass micropipette. An intensity-modulated optically trapped bead positioned as a force sensor on the chromatin fiber was used to measure chromatin local fluidity (as inferred from the phase standard deviation of the bead oscillating in close contact to the chromatin fiber).	An initial increase in the local fluidity (triggered by tension or enzymatic digestion of the histone tails) preceded chromatin decompaction, suggesting possible mechanisms by which chromatin-remodeling factors access regulatory sites.
[43]	Using OT, individual DNA duplexes containing a uniquely positioned nucleosome flanked by long segments of DNA were unzipped to probe histone-DNA interactions and SWI/SNF remodeling activity (setup modified from that shown in Figure 2a).	Nucleosomes remodeled by SWI/SNF were moved bidirectionally with a characteristic distance of motion of ∼28 bp per remodeling event.
[44]	OT were used to examine the force-induced dynamic behavior of a single nucleosome reconstituted on the 601 positioning sequence (Figure 2b).	Nucleosome unravelled in at least two major stages: the first attributed to unravelling of the first (outer) DNA wrap around the histone octamer, the second to the inner DNA wrap.
[45]	OT were used to monitor the activity of RSC and SWI/SNF remodeling factors on single nucleosomal templates made from DNA containing 5–9 tandem repeats of the 601 positioning sequence (Figure 2b).	Remodelers translocated along DNA at ∼13 bp/s and generated forces up to ∼12 pN, producing DNA loops of 20–1200 bp (average ∼100 bp). This behavior differed significantly from that observed on bare DNA [46], suggesting a nucleosome-specific activity of RSC and other remodeling factors of the Swi2/Snf2 family.
[47]	OT were used to unzip individual DNA duplexes containing a uniquely positioned nucleosome (setup modified from that shown in Figure 2a).	A detailed map of histone-DNA interactions was obtained to near bp resolution, revealing a ∼5 bp periodicity superimposed by three regions of strong interactions, the strongest being at the dyad. Unzipping up to the dyad allowed recovery of a canonical nucleosome upon relaxation of the DNA, but unzipping beyond the dyad resulted in irreversible removal of the histone octamer from DNA.
[48]	OT were used to study the force-extension behavior of alpha-satellite DNA from African green monkey, naked or organized as nucleosomes (Figure 2b).	Nucleosomes were disrupted at higher forces as compared with random DNA nucleosomes, suggesting that structural properties of alpha-satellite DNA are responsible for the relatively higher mechanical stability of African green monkey centromeric heterochromatin.
[49]	OT were used to follow individual RNA polymerase II complexes as they transcribe a piece of DNA wrapped in a nucleosome (Figure 2b, but both beads are held by a laser).	The presence of a nucleosome locally increased pause density, slowed recovery from the pause, and reduced pause-free velocity of Pol II. Transcription through a nucleosome seems to involve transfer of the core histones behind the transcribing polymerase via a transient DNA loop.

***Magnetic Tweezers (MT) experiments***		
[30]	MT were used to study chaperone-mediated chromatin assembly/disassembly on single λ phage DNA molecules in real time (Figure 2d).	The rate of assembly strongly depended on the exerted force, with almost total inhibition at forces >10 pN. During assembly at high forces, occasional abrupt increases in fiber length were observed, clearly suggesting reversibility of the assembly process.
[46]	MT were used to monitor the extension of a single DNA molecule at low force (∼ 0.3 pN) in the presence of RSC remodeling factor (Figure 2c).	RSC causes transient shortening of DNA resulting from the formation of a negatively supercoiled loop. AFM images confirmed this model. *Only naked DNAs*, *no nucleosomal susbtrates*, *were tested in this study.*
[31]	MT were used to study the mechanical response to torsion of single nucleosome arrays reconstituted on 36 tandem repeats of a 5S sequence (Figure 2c).	Nucleosome arrays can reversibly accommodate a large amount of supercoiling without much change in length.
[50,51]	MT were used to study the interaction between purified histones and a DNA molecule under tension.	Tension determines the rate of DNA condensation. The time course of compaction was exponential at low histone concentration and became sigmoidal at high concentrations, reflecting a cooperative loading of histones onto DNA. Under large forces, histone-DNA complexes were disrupted in a discrete manner with a step size of ∼60 nm.
[52]	MT were used to study assembly of chromatin on single DNA molecules incubated in *Xenopus* egg extracts (Figure 2b, with a magnetic trap replacing the optical trap).	Force-induced disassembly and opening-closing fluctuations were observed, with a strong dependence on ATP, suggesting that ATP hydrolysis plays a major role in nucleosome rearrangements *in vivo*.
[53]	MT were used to study the mechanical response to torsion of single nucleosomes or tetrasome arrays reconstituted on tandem repeats of a 5S sequence (Figure 2c).	Nucleosome fibers submitted to large positive torsion transiently trapped one positive turn per nucleosome, reflecting a chiral transition of the particle to a metastable, right-handed form (interpretation based on the existence of the previously documented right-handed tetrasome).
[54]	MT were used to reveal molecular interactions at sub-pN forces within sub-saturated (4 ± 3 nucleosomes on 17 tandem repeats of a 5S sequence) reconstituted chromatin fibers (Figure 2c).	When small (∼1 μm) beads are used, the hysteresis caused by viscous drag on the magnetic bead is sufficiently reduced to reveal individual interactions between nucleosomes.
[55]	MT were used to probe the mechanical properties of 167 and 197 bp repeat length arrays of 25 nucleosomes (Figure 2c).	At forces up to 4 pN, the 30-nm fiber stretches like a Hookian spring, resulting in a three-fold extension. Together with a high nucleosome-nucleosome stacking energy, this was interpreted as supporting a solenoid as the underlying topology of the 30-nm fiber (see section 3.4 below). Linker histones do not affect the length or stiffness of the fiber, but stabilize its folding. Fibers with a nucleosome repeat length of 167 bp are stiffer, consistent with a two-start helical arrangement.
[56]	MT were used to measure the force-induced unwrapping of DNA from a single nucleosome (Figure 2c).	Hidden Markov analysis, adopted for the nonlinear force-extension of DNA, can readily resolve unwrapping events that are significantly smaller than the Brownian fluctuations.
[57]	Nucleosome assembly was conducted on single topologically-constrained DNA tethers using chicken erythrocyte core histones and Nap1, under constant low force (Figure 2c). In some experiments, rotation of the external magnetic field was used to relieve the compensatory positive stress presumably accumulating during assembly.	Only partial assembly was observed on the topologically-constrained tethers, whereas much more complete assembly occurred on nicked tethers or on tethers whose superhelical stress was mechanically relieved during the assembly process. The positive supercoiling density that stalled assembly was estimated at 0.025–0.051.

**Table 2.** Some approximate chromatin mechanical parameters.

**Elastic constants**		**Bending persistence length (A)**	**Torsional persistence length (C)**	**Stretching modulus (σ)**
	DNA	50 nm	100 nm	1,100 pN
	Chromatin	30–200 nm	5 nm	5–8 pN
**Destabilizing forces**	Chromatin fiber unfolding (compact 30 nm fiber to “beads on a string”)			<5 pN
	Nucleosome disruption (DNA unwrapping from the surface of histone octamer)			20 pN
